# Genome Sequences of Microbacteriophages Zada and Ioannes

**DOI:** 10.1128/MRA.01012-20

**Published:** 2020-11-05

**Authors:** Razan El Yaman, Jayla S. Anderson, Tania M. Anderson, Michael A. Avalos, Sheku K. Bangurah, Ken B. Dada, Kiefer R. Degener, Mohammad N. Hadeed, Leen H. Issa, Akhteyar S. Jaeran, Katelynn M. Kowalski, Yamere T. Lloyd, Demitra P. Loucopoulos, Vanessa J. Manzo, Nicolas M. Nunez, Andrea M. Sandoval, Semaj Shelton, Steven M. Taddei, Ali A. Zamat, Stephanie B. Conant, Jonathan S. Finkel, Jacob D. Kagey

**Affiliations:** a Department of Biology, University of Detroit Mercy, Detroit, Michigan, USA; b ReBUILDetroit, University of Detroit Mercy, Detroit, Michigan, USA; c Department of Mechanical Engineering, University of Detroit Mercy, Detroit, Michigan, USA; Queens College

## Abstract

Microbacteriophages Zada and Ioannes were isolated from soil and characterized. Genomes were then sequenced and annotated. This was done using the host bacterium Microbacterium foliorum. Zada and Ioannes are both lytic phages with a *Siphoviridae* morphotype.

## ANNOUNCEMENT

Microbacteriophages Zada and Ioannes were extracted and sequenced from the host Microbacterium foliorum. M. foliorum is a commonly used phage host that, to date, has been used to identify 2,197 phages, with 281 sequenced as part of the Science Education Alliance-Phage Hunters Advancing Genomics and Evolutionary Science (SEA-PHAGES) program (1). Zada and Ioannes were extracted at the University of Detroit Mercy in 2018 as part of the SEA-PHAGES Research Coordination Network (2). Both were discovered in soil samples from southeastern Michigan. The M. foliorum used during the isolation of Zada and Ioannes was grown in peptone-yeast-calcium agar at 30°C (3). Zada was isolated using direct plating, while Ioannes was isolated using a different technique of enrichment plating. Zada and Ioannes were characterized as *Siphoviridae* lytic phages based on phage structure, plaque morphology, and sequence similarity to previously characterized phages. Plaque purification was carried out for two rounds via replating to ensure consistent plaque morphology and the presence of a single phage. Phages were then expanded to high titer concentrations for DNA isolation using the Wizard DNA cleanup kit (Promega). Isolated DNA was sent to the University of Pittsburgh for sequencing using the MiSeq (v3) Illumina sequencing platform. Libraries were created using the NEBNext Ultra II kit. Genomes were *de novo* assembled from raw reads using Newbler and Consed (v29) (4, 5), and genome termini were determined as described previously using the Pileup Analysis Using Starts and Ends (PAUSE) program (6, 7). Genome annotation included the identification of all protein-coding and tRNA genes. Open reading frames (ORFs) and predicted protein functions were identified using DNA Master (v5.22.3) (8), Glimmer (v3.02) (9), GeneMark (v2.5) (10), Starterator (8), Phamerator (v3) (11), hhPred (v2.07) (12), and BLASTp (v2.7.1) (13). For annotation purposes, an E value of 10e−4 was used as a cutoff value for hhPred and BLASTp, as previously done in these types of annotations (14). Default parameters were used for all other software unless otherwise specified. Based on genomic organization and sequence similarity to previously annotated and characterized phages, Zada and Ioannes were classified in the subcluster EA1 (15).

Zada was identified as a lytic phage of *Siphoviridae* morphology based on clear plaque formation, electron microscopic imaging, and a lack of any common lysogeny genes, such as integrase (16). Zada has a genome size of 41,814 bp and was sequenced with a coverage of 841-fold, the read length was 150 bp, and 246,839 spots were sequenced. Based on the genome sequence, Zada was grouped in the EA1 subcluster. Phage Ioannes had characteristics similar to those of Zada (lytic, *Siphoviridae,* and lack of integrase), despite being isolated with the enrichment plating method. Ioannes has a genome size of 41,879 bp and was sequenced with 1,278-fold coverage, the read length was 150 bp, and 377,688 spots were sequenced. Ioannes was also classified as an EA1 subcluster phage on the basis of sequence similarity (17). For both phages, the genomic size and organization are in line with those of similar phages found within this phage subcluster (15). Genomes were compared via BLAST and were found to have 98% genomic identity to each other (1).

For both genomes (Zada and Ioannes), all genes were annotated by two independent groups of student annotators. Student annotators used the aforementioned programs to annotate each gene, and any differences in annotations between the two independent groups were reconciled. Following student annotation, two rounds of quality control were performed at the University of Detroit Mercy, with a subsequent round with the SEA-PHAGES quality control team checking all genomes for completeness in annotations. We believe these to be complete genome annotations for Zada and Ioannes.

### Data availability.

GenBank and SRA accession numbers are listed in Table 1.

**TABLE 1:** Features of the microbacteriophages

Phage name	GenBank accession no.	SRA accession no.	Length (bp)	G+C content (%)	No. of ORFs	No. of tRNAs	Cluster^*a*^	Life cycle	Location of isolation
Zada	MT310856	SRX8358934	41,814	63.5	64	0	EA1	Lytic	Dearborn, MI, USA
Ioannes	MN735430	SRX8358933	41,879	63.4	63	0	EA1	Lytic	Ann Arbor, MI, USA

a Cluster, a grouping of similar bacteriophages based on genome sequence similarities.
